# Pricing New Drugs for COVID-19 in the Pharmaceutical Industry: Insights From the Chinese Medical Insurance

**DOI:** 10.34172/ijhpm.2023.7972

**Published:** 2023-06-18

**Authors:** Jinmiao Lu, Xiaohua Ying, Zhiping Li

**Affiliations:** ^1^Department of Clinical Pharmacy, National Children’s Medical Center, Children’s Hospital of Fudan University, Shanghai, China; ^2^NHC Key Laboratory of Health Technology Assessment, Department of Health Economics, School of Public Health, Fudan University, Shanghai, China

## Dear Editor,

 The zero-price conundrum in drug pricing is the dilemma pharmaceutical companies face in pricing their drugs.^1^ They must balance accessibility and affordability for consumers with profitability to cover research and development costs. Government and insurance restrictions on drug prices can make profitability difficult. Conversely, recent research indicates that the pharmaceutical industry’s “Financialization” has resulted in significant allocations of funds towards share buybacks and dividends, surpassing expenditures on research and development. This trend may contribute to the elevated prices of drugs.^2^

 Historically, Chinese drug pricing has undergone three phases: State-set pricing, government-suggested pricing, and company-determined pricing. Since 2015, innovative drug pricing has been the responsibility of companies. These drugs tend to be expensive in a fragile healthcare payment system. On January 6, 2023, the China Medical Security Administration released its first official guideline on pricing innovative drugs for COVID-19 treatments.^3^ This guide is essential for the public to understand how new drugs are priced. China’s health insurance policy requires a three-step price-setting process for public medicines: Expert recommendation, official review, and regular reassessment (Figure). We examine future trends and price-restriction measures for drug inclusion in China’s medical insurance system.

**Figure:**
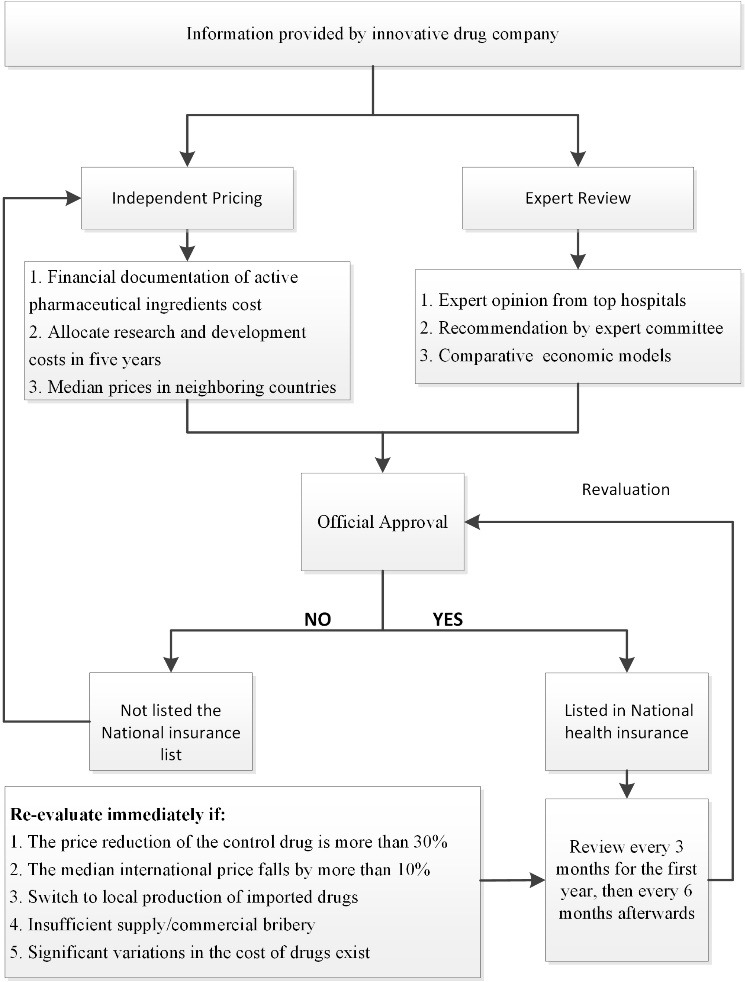
The Flowchart of National Health Insurance Drug Pricing.

 In China, medical institutions are the main buyers and sellers of drugs for medical insurance funds. These facilities with access to Medicare drugs include all government hospitals and public community health centers. These medical institutions’ drug prices are all ex-factory, making it easier for policy-makers to control. To avoid bribery, the cost of pharmaceutical sales must be within 110% of the average of listed companies. These enterprises are listed in China’s Shanghai Stock Exchange section of the Science and Technology Innovation Board in all drug manufacturers. In contrast, drug pricing in the United States is complex and not easily understood, with different prices for manufacturers and patients.^4^

## Hospital Expert, Committee Recommendation, and Economic Evaluation

 The guidelines require drug companies to provide three recommendations: senior expert advice from hospitals, guidance from the association’s Panel on drug prices, and health technology assessments.^5^ These opinions include the treatment value, clinical demand, procurement price expectations, and other aspects of therapeutic drugs. For example, clinical experts need to consider multiple factors when providing their views on pricing for new drugs, including the severity of the disease, patient demand, treatment efficacy, and available alternative medications. Additionally, they should consider the economic affordability and public interest in the prescription to balance patient interests, the sustainability of the healthcare system, and incentives for research and development, ensuring the pricing is reasonable and fair. Typically, the evaluation results are expressed through a scoring system. This score indicates the degree of expert recommendation, such as solid recommendation, general recommendation, no recommendation, or objection.

 The results of the guidelines require that the price quoted for new drugs be no higher than 130% of the procurement price expected in the submissions. National pharmaceutical industry associations need to set up drug pricing expert groups and invite pharmaceutical, clinical, drug economics, pricing, and other experts to participate. The drug treatment subcommittee has a similar composition to the multidisciplinary subcommittee in the United States.^6^ The primary members of the interdisciplinary subcommittee include physicians, nurses, and pharmacists.

 Pharmacoeconomic evaluation results require that a new drug’s combined cost be lower than or equal to the price of a course of treatment for a controlled drug. The total cost of a new drug encompasses research and development costs, production costs, registration and approval costs, sales and marketing costs, intellectual property expenses, supply chain management costs, and regulatory compliance costs, which can vary depending on specific circumstances, drugs, and regions. The combination of these costs shall prevail mainly to the manufacturers provided by the financial vouchers. The results of a price control study have shown that new drugs are not introduced in countries where the expected price is low, or the expected market size is small, or are released late.^7^ However, taking cost-effectiveness as the coverage criterion can significantly save medical expenses.

## Official Reviews and Regular Reassessments Strategy

 Companies must review their drug prices every three months after launch and every six months after the first year. The drug manufacturer must also promptly adjust their prices in the event of (1) a 30% or more significant decrease in the control drug price through economic evaluation and innovation, (2) a drop in the median international price of more than 10%, (3) conversion from imported to local production, (4) excessive pricing caused by severe price dishonesty, and (5) significant changes in factors determining price, including an increase in active pharmaceutical ingredient price.

 In conclusion, balancing cost-effectiveness and medical needs while maintaining financial responsibility is vital in determining public drug prices. The goal is to balance innovation and economic stability in healthcare. China’s drug pricing regulations may limit new drug introduction, but they lower costs and increase accessibility for the public.

## Ethical issues

 Not applicable.

## Competing interests

 Authors declare that they have no competing interests.
